# Generation and quality control of lipidomics data for the alzheimer’s disease neuroimaging initiative cohort

**DOI:** 10.1038/sdata.2018.263

**Published:** 2018-11-20

**Authors:** Dinesh Kumar Barupal, Sili Fan, Benjamin Wancewicz, Tomas Cajka, Michael Sa, Megan R. Showalter, Rebecca Baillie, Jessica D. Tenenbaum, Gregory Louie, Alzheimer’s Disease Neuroimaging Initiative, Alzheimer’s Disease Metabolomics Consortium, Rima Kaddurah-Daouk, Oliver Fiehn

**Affiliations:** 1 NIH-West Coast Metabolomics Center, University of California, Davis, 451 Health Sciences Drive, Davis, California 95616, United States; 2 Rosa & Co LLC, San Carlos, CA, 94070 USA; 3 Department of Biostatistics and Bioinformatics, Duke University, Durham, NC, 27708 USA; 4 Department of Psychiatry and Behavioral Sciences; Department of Medicine and the Duke Institute for Brain Sciences, Duke University, Durham, NC, 27708 USA; 5 Department of Biochemistry, Faculty of Sciences, King Abdulaziz University, Jeddah 21589, Saudi Arabia

**Keywords:** Diagnostic markers, Alzheimer's disease, Epidemiology, Metabolomics

## Abstract

Alzheimer’s disease (AD) is a major public health priority with a large socioeconomic burden and complex etiology. The Alzheimer Disease Metabolomics Consortium (ADMC) and the Alzheimer Disease Neuroimaging Initiative (ADNI) aim to gain new biological insights in the disease etiology. We report here an untargeted lipidomics of serum specimens of 806 subjects within the ADNI1 cohort (188 AD, 392 mild cognitive impairment and 226 cognitively normal subjects) along with 83 quality control samples. Lipids were detected and measured using an ultra-high-performance liquid chromatography quadruple/time-of-flight mass spectrometry (UHPLC-QTOF MS) instrument operated in both negative and positive electrospray ionization modes. The dataset includes a total 513 unique lipid species out of which 341 are known lipids. For over 95% of the detected lipids, a relative standard deviation of better than 20% was achieved in the quality control samples, indicating high technical reproducibility. Association modeling of this dataset and available clinical, metabolomics and drug-use data will provide novel insights into the AD etiology. These datasets are available at the ADNI repository at http://adni.loni.usc.edu/

## Background and Summary

In the past century, human life expectancy has improved considerably, but has also lead to an increased in the number of patients of chronic diseases such as Alzheimer’s Disease (AD). AD is a neurodegenerative disease that irreversibly destroys the normal ability of the brain to access memories and make decisions^1^, and the affected person becomes demented with a decreasing quality of life.

The causes of the onset and progression of AD have not yet been identified but differ from normal aging. While AD is associated with the presence of tau neurofibrillary tangles and amyloid beta (Aβ) plaques in the post-mortem brain^2–4^, many other changes in both central and peripheral physiology have been linked to the disease^5^. Previous research suggests that the disruption of lipid metabolism is part of the pathophysiology of AD^6^. Lipids have clear roles in amyloidogenesis, neurotransmission, oxidative stress, and apoptosis, all thought to be tied to AD progression. Both central and peripheral lipid metabolism is altered in AD subjects and lipid metabolites in the cerebral spinal fluid and blood may provide information on the metabolic pathways contributing to AD pathophysiology.

While tau and Aβ plaques are prominent among the recognized AD markers^7,8^, it is important to identify other biochemical changes which are related to disease development and progression. By identifying changes in other biological pathways associated with AD^9^, we suggest new targets to treat or biomarkers to diagnose this disease. Our limited understanding of AD is one of the main reasons that we cannot stop the progression of this disease^10^. Identification of biomarker and druggable target specific genetic, molecular, and cellular mechanisms may help to prevent AD progression. However, results have been unsatisfying to date^11,12^.

Progress in analytical chemistry now allows for the rapid and reproducible screen for hundreds of lipid molecules from triacylglycerol, sphingomyelins, lysophosphatidylcholine, phosphatidylcholine, cholesteryl esters, fatty acids and other lipid classes^13^, now referred to as lipidomics^13–15^. Lipidomics has been frequently used to associate blood lipid changes with chronic diseases such as cardiovascular disease, depression and AD^6,16^. Today, up to 800 different lipid molecules can be measured in human blood using liquid chromatography-high resolution mass spectrometry^17,18^.

Lipids are a major chemical group that contributes significantly to the brain functioning^19^. A range of studies suggest the role of hypometabolism in the progression of AD^20–22^. Diabetes and insulin resistance also increase the risk of AD in older age^23–28^. Therefore, we hypothesized that the onset of AD may be associated with body’s lipid metabolism. To test this hypothesis, blood lipid’s levels can be used to link the lipid metabolism with AD. Previous studies support this hypothesis^29–31^, but evidence needs to be validated and solidified for specific lipid classes. The link of blood lipids with onset and progression of AD needs to be tested in large epidemiological studies so that the effect of confounding factors such as aging, medication and gender can be taken into account while investigating the associations of AD with lipids.

The Alzheimer’s Disease Neuroimaging Initiative (ADNI) was initiated in 2004 to facilitate the validation of biomarkers for clinical studies on AD^32^. ADNI^32,33^ provides a unified online platform to researchers around the globe so that they can define the progression of Alzheimer’s disease. The platform enables the collection, validation and use of data such as magnetic resonance imaging and positron emission tomography images, genetics, cognitive tests, cerebral spinal fluid and blood biomarkers from thousands of individuals as predictors for the disease^34^. A targeted metabolomics of ADNI1 cohort serum specimens has been completed using the Biocrates AbsoluteIDQ p180 kit^35^. In the dataset, 121 lipids species were measured using the flow-injection analysis-MS/MS and were reported using their semi-quantitative intensities. The biocrates targeted method quantifies four lipid classes – acyl carnitines, lyso phosphatidylcholine, phosphatidylcholines and sphingomyelins using the direction flow ionization mass spectrometry. This analytical method suffers by the presence of isomeric lipid species in the sample therefore one signal can represent several lipid species. Whereas a liquid chromatography (LC) based separation can separate these isomeric lipid species and give multiple signals. In the presented data descriptor, up to 13 lipid classes including triacylglycerols, ceramides, phosphoethanolamine, lysophosphoethanolamine phosphoinositols, glyceroceramides, diacylglycerols, cholesterol esters were covered. Several lipids in these classes are not covered by other biochemical datasets available at the ADNI website^35^. For many lipids we have the acyl chain information available using their MS/MS spectra. For this consortium, a comprehensive and untargeted lipidomics dataset is presented here for the study the association body’s lipid metabolism with AD and its clinical features. The NIH-West Coast Metabolomics Center has developed a liquid chromatography and mass spectrometry based lipidomics assay of blood specimens^36–40^. Here, we used a UHPLC-QTOF mass spectrometer to detect, identify and measure lipids in blood samples from 806 individuals from the ADNI study and describe the details of the samples preparation, data generation, quality controls, data filtering and normalization. The ADNI repository hosts the whole genome sequencing, mitochondrial DNA sequencing, genotyping array, magnetic resonance imaging, positron emission tomography scans, blood and cerebrospinal fluid protein and peptide biomarker panel, medication use, anthropometric measurements, metabolomics and cognitive measurements for the recruited participants. The primary use to the data descriptor is to discover associations between serum lipid levels and these available information from the ADNI repository. To find these associations, linear or logistic regression models need to be computed while adjusting for key confounding variables such as age, gender or body mass index. Significant associations may indicate which lipid metabolic pathways can be implicated in the AD etiology.

## Methods

### Alzheimer’s Disease Neuroimaging Initiative (ADNI) cohort

The ADNI data repository (http://adni.loni.ucla.edu/) supplied all the demographic information, neuropsychological and clinical assessment data, and diagnostic information used for this study and this information has been published previously^5^. Prior Institutional Review Board approval was obtained at each participating institution and written informed consent was obtained for all participants. Information about ADNI can be found in Petersen et al. 2010 and at http://www.adni-info.org/.^41^ Key clinical and demographic variables for the ADNI1 participants used in this study are summarized in Table 1 and are available through Synapse (see Table 2). Note that ADNI data collection is ongoing, so variables on the Laboratory of Neuro Imaging (LONI) repository may have been updated since the subset used in this analysis was downloaded. Access to ADNI data can be requested at http://adni.loni.usc.edu/data-samples/access-data/ after signing a data use agreement.

### Serum specimen collection and sample management

Fasting blood samples from the baseline visit were included in the study (69 non-fasting samples were excluded post analysis). Blood samples were collected in two bar-coded 10 mL red-top plastic Vacutainer blood tubes, blood was allowed to clot for 30 min followed by a 15 min centrifugation at 3000 revolutions per minute (1500 relative centrifugal force) as described in the ADNI standard operating procedures (www.adni-info.org). The serum was transferred into a bar-coded 13 mL polypropylene transfer tube, capped and frozen in dry ice. Samples were shipped overnight to the ADNI biomarker core laboratory at the University of Pennsylvania Medical Center. In the core facility, samples were thawed and aliquoted to 0.5 mL samples, and then subsequently aliquoted once more for individual laboratory analyses. A 20 μL sample aliquot was delivered for analysis with the lipidomics platform at the West Coast Metabolomics Center at UC Davis. Samples were randomized before shipping to the analysis laboratories. As a positive control, 20 samples replicated and inserted randomly across the analysis sequence.

### Equipment and consumables for sample preparation and Liquid chromatography-mass spectrometry (LC-MS) analysis

Equipment and consumables used for the sample preparation and the LC-MS data acquisition were - Agilent 1290 Infinity UHPLC/6530 QTOF MS and Agilent 1290 Infinity UHPLC/6550 QTOF MS, Acquity charged surface hybrid technology (CSH) C18 2.1×100 mm, 1.7 μm column (Waters, cat. no. 186005297) and Acquity VanGuard CSH C18 2.1×5 mm, 1.7 μm pre-column (Waters, cat. no. 186005303), centrifuge (5415D, Eppendorf), calibrated pipettes 10-200 μL and 100-1000 μL, Eppendorf tubes (1.5 mL, uncolored, cat. no. 022363204), MiniVortexer (VWR), Orbital Mixing Chilling/Heating Plate (Torrey Pines Scientific Instruments), speed vacuum concentration system (Labconco Centrivap) with a cold trap, Agilent tune mix (Agilent, cat. no. G1969-85000), acetonitrile (ACN) (J.T. Baker, cat. no. 9829-03), formic acid (Fluka, cat. no. 94318-250mL-F), ammonium formate (Fluka, cat. no. 70221-25G-F), isopropanol (Sigma–Aldrich, cat. no. 34965-4 × 2.5 L), LC-MS grade water (Fisher, cat. no. W6-4), methyl *tert*-butyl ether (MTBE) (Sigma–Aldrich, cat. no. 34875-100 ML), methanol (Fisher, cat. no. A456-4), toluene (Sigma–Aldrich, cat. no. 179418-500 ML), ammonium acetate (Sigma–Aldrich, cat. no. A7330-100G).

### Internal standards

Cholesteryl ester (22:1) was purchased from Nu-Chek (cat. no. CH-848). Avanti Polar Lipids supplied the pure standards for phosphatidylethanolamine (17:0/17:0) (cat. no. 830756 P), 1,2-diheptadecanoyl-sn-glycero-3-phospho-(1’-rac-glycerol) (17:0/17:0) (cat. no. 830456 P), 1-heptadecanoyl-2-hydroxy-sn-glycero-3-phosphocholine) (cat. no. 855676), C17 sphingosine (cat. no. 860640), C17 ceramide (d18:1/17:0) (cat. no. 860517), sphingomyelin (d18:1/17:0) (cat. no. 860585), PC (12:0/13:0) (cat. no. LM-1000), *d*_7_-cholesterol (cat. no. LM-4100), *d*_5_-triacylglycerol (17:0/17:1/17:0) (cat. no. 110544), diacylglycerol (12:0/12:0/0:0) (cat. no. 110611), diacylglycerol (18:1/2:0/0:0) (cat. no. 800100), monoacylglycerol (17:0/0:0/0:0) (cat. no. 110607) and phosphatidylethanolamine (17:1/0:0) (cat. no. 110699). *d*_3_-Palmitic acid (cat. no. D-1655) was supplied by CDN isotopes. For the extraction, methanol containing a mixture of internal standards (without CE (22:1)) was prepared, while MTBE containing cholesteryl ester (22:1) was used. These internal standards were used only for the retention time recalibration. Signals across samples were corrected by a bioreclamation quality control plasma based normalization method.

### Extraction of lipids from the blood samples

Extraction solvents methanol (MeOH) and MTBE were sonicated for 5 min, then stored at -20 °C prior to use. Serum and plasma (QC) samples were thawed at room temperature and placed on ice. Samples were vortexed for about 5 s to obtain homogenized samples. For each sample, a 20 μL aliquot was transferred to an Eppendorf tube and 225 μL of -20 °C cold MeOH containing a mixture of internal standards was added. Samples were vortexed for 10 s. Then 750 μL of -20°C MTBE with CE (22:1) was added, vortexed for 10 s and shaken for 6 min at 4 °C. LC-MS grade water (188 μL) was added and the mixture was vortexed for 20 s. Samples were centrifuged for 2 min at 14,000 rcf. The upper organic phase was divided into two aliquots of 350 μL. Solvents were removed under vacuum to complete dryness in a speed-vacuum dryer. One aliquot was kept at -20 °C as backup, the other dried-down sample was re-suspended in 100 μL of a MeOH/toluene (9:1, v/v) mixture containing an internal standard 12-[[(cyclohexylamino)carbonyl]amino]-dodecanoic acid (CUDA) (Cayman Chemical, cat. no. 10007923) and vortexed for 15 s. For quality controls and for normalizations, one blank negative control extraction was prepared per 10 actual samples from empty Eppendorf tubes as starting material, in addition to one Bioreclamation plasma (BioreclamationIVT, cat. no. HMPLEDTA) QC sample per 10 actual study samples.

### UPLC-QTOF MS analysis

Samples were analyzed in four batches. Before each batch, the ion source was cleaned and the VanGuard pre-column was replaced. Extracts were separated using a charged surface hybrid (CSH) C_18_ column (Waters). Data were acquired using a single charged surface hybrid 2.1 mm id × 100 mm length, 1.7 μm particle C_18_ column (Waters). The mass spectrometer was tuned before each batch as per the vendor’s instructions using Agilent tune mix (mass resolving power ~10,000 Full width at half maximum (FWHM) and ~20,000 FWHM for the Agilent 6530 and 6550, respectively). A reference solution of purine and HP-0921 (*m*/*z* 121.0509, *m*/*z* 922.0098 in electrospray ionization (ESI) ( + ) and *m*/*z* 119.0360 and *m*/*z* 980.0164 (acetate adducts) in ESI(–)) was used to correct small mass drifts during the acquisition. Mobile phase A (60:40 ACN:water + 10 mM ammonium formate + 0.1% formic acid) was prepared by mixing 600 mL ACN, 400 mL water, 1 mL formic acid and 630 mg of ammonium formate. Mobile phase B solvent (90:10 IPA:ACN + 10 mM ammonium formate + 0.1% formic acid) was prepared by mixing 900 mL IPA, 100 mL acetonitrile, 1 mL formic acid, 630 mg ammonium formate previously dissolved in 1 mL of H_2_O. Both solvents were mixed and sonicated for 10 min (twice) before their use. For ESI(–) the composition of mobile phases was identical but 10 mM ammonium acetate (771 mg per 1 L) was used instead as modifier. Before starting each batch of sample analyses, one empty injection, five solvent injections (MeOH/toluene 9:1 with CUDA internal standard), two extraction blanks and two Bioreclamation pool plasma samples were injected. Instrument performance was monitored for column backpressure and signal intensities of the reference compounds. The quadrupole/time-of-flight (QTOF) mass spectrometers are operated with electrospray ionization (ESI) performing full scan in the mass range m/z 65–1700 in positive (Agilent 6530, equipped with a JetStreamSource) and negative (Agilent 6550, equipped with a dual JetStream Source) modes producing both unique and complementary spectra. Instrument parameters were as follows for the ESI ( + ) mode – gas temperature 325 °C, gas flow 8 l/min, nebulizer 35 psig, sheath gas temperature 350 °C, sheath gas flow 11, capillary voltage 3500 V, nozzle voltage 1000 V, fragmentor voltage 120 V and skimmer 65 V. In negative ion mode, gas temperature 200°C, gas flow 14 l/min, fragmentor 175 V, with the other parameters identical to positive ion mode. Data are collected in centroid mode at a rate of 2 scans per second. Injection volume was 1.7 μL for the positive mode and 5 μL for the negative mode. The liquid chromatography gradient used a 0.6 mL/min linear velocity flow rate. The gradient started at 15% B, ramped to 30% at 2 min, 48% at 2.5 min, 82% at 11 min, 99% at 11.5 min and kept at 99% B until 12 min before ramping down to 15% B at 12.1 min which was kept isocratic until 15 min to equilibrate the column. The total run time was 15 min. Acquisition speed was 2 spectra/s and the mass range was *m*/*z* 60-1700. After every ten cohort samples, one Bioreclamation pooled plasma QC sample was analyzed. These samples were used for correction of the batch effect using a local polynomial regression fitting (LOESS) polynomial regression based normalization approach. All the acquired raw LC-MS data files are provided in the data record (ADNI1_LIPIDOMICS_RAW_DATA_ESI_POS.zip (Data Citation 1) & ADNI1_LIPIDOMICS_RAW_DATA_ESI_NEG.zip) (Data Citation 2) (see Table 2).

Raw LCMS data files were provided in the vendor format (.d) to avoid any data loss during the file conversion. The format contains the information such as time-stamps of the data acquisition, instrument parameters and the liquid chromatography gradient. These information are saved in readable xml files in the vendor format (.d) directory. The format also has the back-pressure profile on the LC system which can be helpful in checking the system stability. These vendor files can be converted to an open format such as mzML using the Proteowizard MSConvert utility so they can be read into other software such as mzMine. Because the conversion will ignore the key parameters such as the back-pressure profile, we decided to provide the raw data in the vendor format.

### Lipid ion database

A database of validated lipids that were routinely detected in blood samples has been compiled at the West Coast Metabolomics Center over the past six years. In this database, annotated lipids are associated with retention time, adducts, *m*/*z* value and InChiKeys, verified by accurate mass, isotope ratio, retention time and MS/MS spectra matching to either commercial lipid standards or to the LipidBlast library^42^. In the database, ESI positive mode had 515 *m*/*z* values and ESI negative mode had 457 *m*/*z* values. Table 3 summarizes the database content. The database had *m*/*z* values for 374 unique known lipids and 25 internal standards. A total 98 lipids had *m*/*z* values in both ESI(–) and ESI( + ) modes. Known unknown lipids are species that have been observed consistently in blood samples from the metabolomics projects at the West Coast Metabolomics Center at the University of California, Davis over last six years however have not been annotated yet. They have only *m*/*z* values and retention time. The database is provided in the data record (wcmc-lipidomics-database.zip).

### LC-MS data processing

Using the ion database as a targeted list, we have extracted the ion chromatograms (EICs) for all the *m*/*z* values using the Agilent MassHunter Qualitative Analysis (7.0) software. We have used a targeted signal extraction strategy for the study. Find by formula (FBF) method in the Agilent Mass Hunter software was used only for extracting ion chromatograms for the *m*/*z* values provided in the ion database. We have extracted ion chromatograph for 1 min before and after the expected retention time for each *m*/*z* value. Since the database already had adduct information, ‘no adduct’ parameter was chosen in FBF settings and only *m*/*z* values were used. EICs were used later to obtain the peak height values for lipids. No peak smoothing and integration method was applied to the EICs. The ion database had retention time information that exactly where the peak maxima for a lipid *m*/*z* value is so we have extracted peak maxima for each peak at the corresponding retention time. For example TG (58:8) [M + NH_4_]^+^ has *m*/*z* value 948.8015 and expected retention time 10.167 min in the ion database. We first extracted the ion profile in the range 10.06-10.26 min and then obtained the max value of that profile as peak maxima for TG (58:8) [M + NH_4_]^+^. All the EICs are provided at the LONI database. Using the Agilent DA Reprocessor offline utility tool, the method was applied to all the raw LCMS data files (.d) in a batch mode. Data processing method files are provided in the data record (adni1-qual-method.zip). The output CSV files are provided in the data record (adni1-lipidomics-eics.zip) in Data Citation 3. These files contain intensities of the ions over the retention time range (retention time ± 1.0 min) for each *m*/*z* value from the target database. These CSV files were imported into R software for further data processing. First the retention time of the internal standard were obtained for each sample. A retention time window of 0.2 min was used for finding the peaks for internal standards. Observed retention times for the internal standards were determined by finding the time point at which the intensity of the *m*/*z* value was maximum within the retention time range. A polynomial regression of second order (quadratic) curve was fitted between the expected and observed retention times of the internal standards. The retention times of the remaining compounds in the target database were recalibrated using the regression model. The updated retention times were used with a window of 0.20 min for each metabolite to extract the *m*/*z* intensities values belonging to that ion from the CSV files. Maximum intensity values within the retention time window were used as the peak height of the target compounds. Extracted targeted peaks for each sample were exported as .txt files. These files were imported into R and merged to form the raw lipidomics dataset. The signal-to-noise ratio was calculated by dividing the detected peak height of a lipid by the median of ion intensities within the retention time range for the m/z value of that lipid. Next, data were first normalized using the LOESS algorithm to remove trends and drifts in the data set within each batch for each individual lipid. Then, a median normalization was applied across all the batches. In order to prevent overfitting, the degree of smoothing for each LOESS curve was determined using leave-one-out cross validation. We used a 5-fold cross validated relative standard deviation (RSD) of the QC samples to measure the performance of the normalization procedure. The scatter plots of the injection order and the signal intensity for individual lipids and the principal component analysis plots of the overall dataset before and after the LOESS correction are provided in the data record(“adni1_loess_results.zip”)

Raw LC-MS data were acquired in an untargeted fashion and have been made available through LONI’s data repository. A targeted signal extraction strategy was used to generate the lipidomics data matrix. First, ion profiles for all *m*/*z* values, 515 in ESI( + ) mode and 457 in ESI(–) mode were extracted from the raw LC-MS data files. For several lipids, more than two ions representing multiple adducts were extracted in both ESI positive and negative modes. Then a data filtering pipeline was used to select one adduct for each lipid with the lowest technical variance in the quality control samples. For example, if lipid X has two adducts (a1 + , a2 + ) in ESI( + ) and two (a1-, a2-) in ESI(–) modes. First, one adduct in each mode was selected using the lowest QC RSD criteria. For example, a1 + and a2- were selected. Then it was determined which one of these two has lowest RSD in QC samples and that was included into the final data matrix and the data dictionary. And if lipid *Y* has one adduct (a1 + ) in ESI( + ) and one (a1-) in ESI(–) modes. For this, it was determined which one of these two has lowest RSD in QC samples. Peak heights of those selected ions were used as a measurement for lipid species in one ionization mode. After filtering out multiple adducts for the same lipid, a total 367 entries in ESI ( + ) and 280 entries in ESI (−) modes files were remained. A total 98 lipids were detected in both modes. To minimize the penalty for multiple hypothesis testing while calculating false discovery rates for regression modelling, duplicate entries for lipids were removed by using the relative standard deviation in the quality control samples. An entry with lowest RSD should be selected for lipids having duplicate entries in the dataset. We have also computed the spearman correlation between peak heights values in both modes for lipids. Out of the total 98 duplicate detection in both mode, 80 (81%) showed a correlation of 0.80 or more, indicating that the pairs belong to same lipid species and have good quality data in both modes. The lower correlation for rest of 12 pairs can be attributed to poor detection in one ionization mode or presence of isomers. Therefore, we have used the RSD in QC samples for all these pairs to decide which lipid should be included in the data matrix that is ready for statistical analysis. Final data matrix had 513 unique lipids species out of which 341 were known lipids and 172 were known unknowns. This final dataset is provided in the data record (ADMC_ADNI1_LIPIDOMICS.csv) in Data Citation 4. Details about the measured lipid species are provided in the data record (ADNI_ADMCLIPIDOMICS_DICT.csv). Raw input matrices for ESI (−) and ESI ( + ) modes and intermediate matrices during the data filtering and merging steps have been provided in the data record (data_processing_matrices.zip). The R-script file describes the steps at which these files were generated during the data processing.

Untargeted lipidomics assays allow a broad survey of lipid classes and changes in their levels. In these assays, lipid’s chromatography peak height or integrated peak area represents its levels in a sample^38,43,44^. As pure chemical standards for every detected lipids species in an untargeted assay are not commercially available, it is not feasible to precisely estimate the absolute molar concentrations of them, therefore, peak heights are used for creating lipidomics data matrices. Statistical analyses of those peak heights across study samples identifies the significant lipid species that associate with an outcome of interest^45^. Use of the QC based LOESS normalization method removed the batch effect in this study so that samples can be compared for statistical purposes within the study. So if a compound has a mean peak height of 25,000 in a disease group and a mean peak height of 75,000 in a control group, these values can reliably be used to determine a 3 fold decrease in levels of the compound in the disease group in comparison to the control group. Since internal standard for every measured lipids are not available, it is not possible to precisely assess the signal linearity or concentration ranges for every lipid. Nevertheless, the Agilent 6530 and 6550 UHPLC-QTOF instruments that were used in the study have a linear dynamic range of up to 3 orders of magnitude. This range can provide reliable signals to identify up to a 100 fold decrease or increase in the levels of a majority of lipids, for example a mean peak height of 25,000 in cases versus 2,500,000 in control samples. Peak height of one compound can only be used to compare its level across samples, it cannot be compared with another compound’s peak height due to different ionization efficiency. For example, if lipid A has a peak height of 25,000 and lipid B has 50,000, this does not mean that lipid B is two times more than lipid A. It is needed that the data matrix need to be scaled and transformed according to the statistical analyses.

Reversed-phase LC column with C18 sorbent and gradient separate lipid species by lipophilicity, thus, it can separate two chemically different lipids even with same mass. However it cannot separate two lipids species having same lipophilicity and mass values. In this case MS/MS spectra collected for particular peak can inform if the peak consists of one or more co-eluting compounds. For example, there can be multiple isobaric lipid species under one chromatographic peak, for example TG (48:0) may consist of TG (16:0/16:0/16:0), TG (18:0/16:0/14:0), TG(18:0/14:0/16:0) and TG(14:0/18:0/16:0), depending on the acyl chains. These isomers may elute at the same time and thus provide just a single peak. To confirm the purity of such peaks, MS/MS spectra were collected for each lipid species and we have confirmed that up to 70% of the peaks have only one lipid species as per matching their MS/MS spectra against LipidBlast library. NIST MS Search software was used to search the MS/MS spectra against LipidBlast library and then each spectrum was manually checked. TG (48:0) has a clean MS/MS spectrum and we have confirmed that it is TG (16:0/16:0/16:0). Rest 30% of the peaks showed mix MS/MS spectra with multiple isobaric lipid possibilities, and for them we have selected the most abundant acyl chains in the MS/MS spectra as acyl chain annotation. Because acyl chain’s sn position could not be determined for several lipids, the naming convention uses “_” instead of “/” for the lipids with multiple acyl chains in the lipidomics dataset.

### Statistical analysis

Principal component analysis, paired *t*-test, histogram and calculations of relative standards deviations were performed in the R programming language version 3.4.0.

### Code availability

R script to generate the lipidomics dataset from the extracted ion chromatograms is provided in data record (adni1-lipidomics-script.r).

## Data records

**Figure 1:**
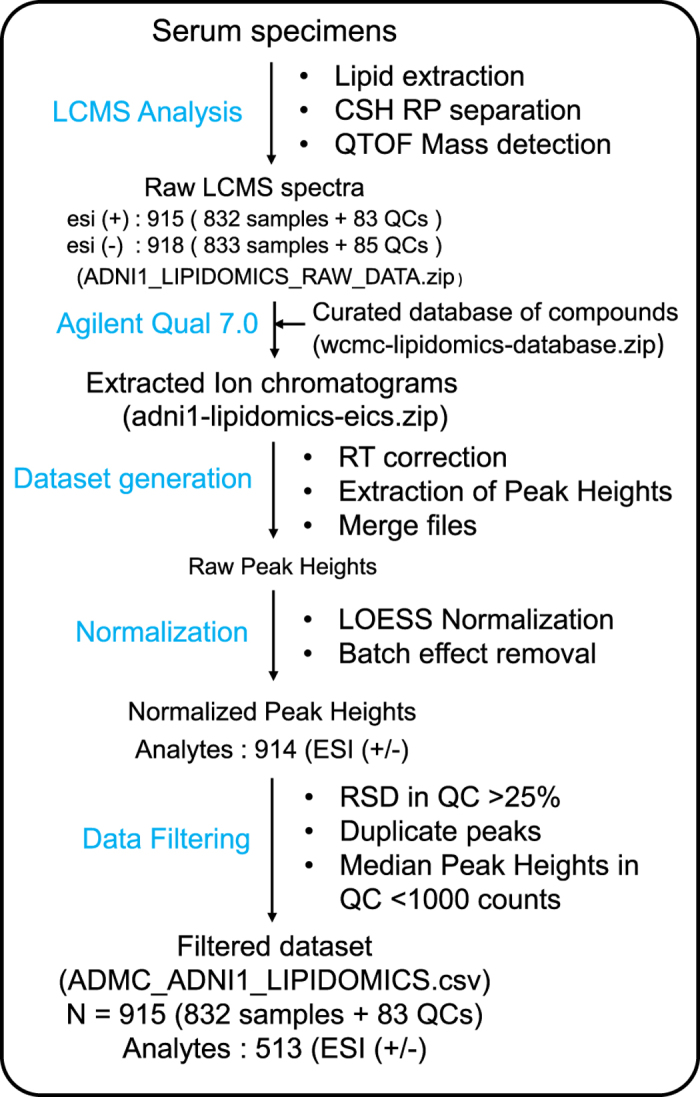
Overview of the lipidomics data generation workflow.

The primary access site for this dataset is through Sage Bionetworks’ Synapse platform: https://www.synapse.org/#!Synapse:syn9993525. Detailed information about ADMC data are provided in https://www.synapse.org/#!Synapse:syn5592519. Input raw LC-MS mass spectra data files (Fig. 1) are found in (Data Citation 1) and (Data Citation 2) for ESI positive and negative mode respectively. Extracted ion chromatograms (Fig. 1) are available at (Data Citation 3). Processed data matrix for association modeling is available at (Data Citation 4). ADNI’s data use agreement prohibits redistribution of ADNI data outside of LONI, so actual data files are hosted by the University of Southern California’s Laboratory of Neuroimaging (LONI). The scripts used for data processing and medication mapping, however, reside in the Synapse platform. Core data files along with associated metadata files, scripts, and supplemental files are listed in Table 2. Note that ADNI requires registration to access the raw LCMS data files and processed data matrices. Researchers may apply for data access at https://ida.loni.usc.edu/collaboration/access/appLicense.jsp.

## Technical validation

**Figure 2:**
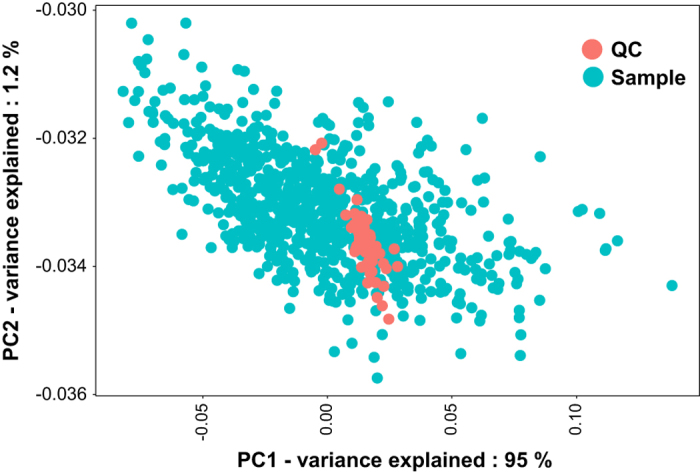
Principal component analysis showing the overall variance among analyzed samples.

For the technical verification of the lipidomics dataset workflow, we analyzed the quantities of internal standards and measured lipids in study samples, duplicate samples and quality control samples. First, we performed a principal component analysis (PCA) of the lipidomics dataset to test for overall lipid and sample variance, for example, to screen for outliers. The PCA score plot showed a larger-variance cluster of human cohort samples and a small-variance cluster of Bioreclamation QC samples (Fig. 2). Of the total variance, 95% was explained by the difference between these two clusters in principal component 1, compared to only 1.2% variance by the next-most important vector, principal component 2. Analysis of sample groups by further principal components did not reveal any impact of batch effects or injection time points, showing that this data set does not contain major injection bias error or other hidden bias groups.

**Figure 3:**
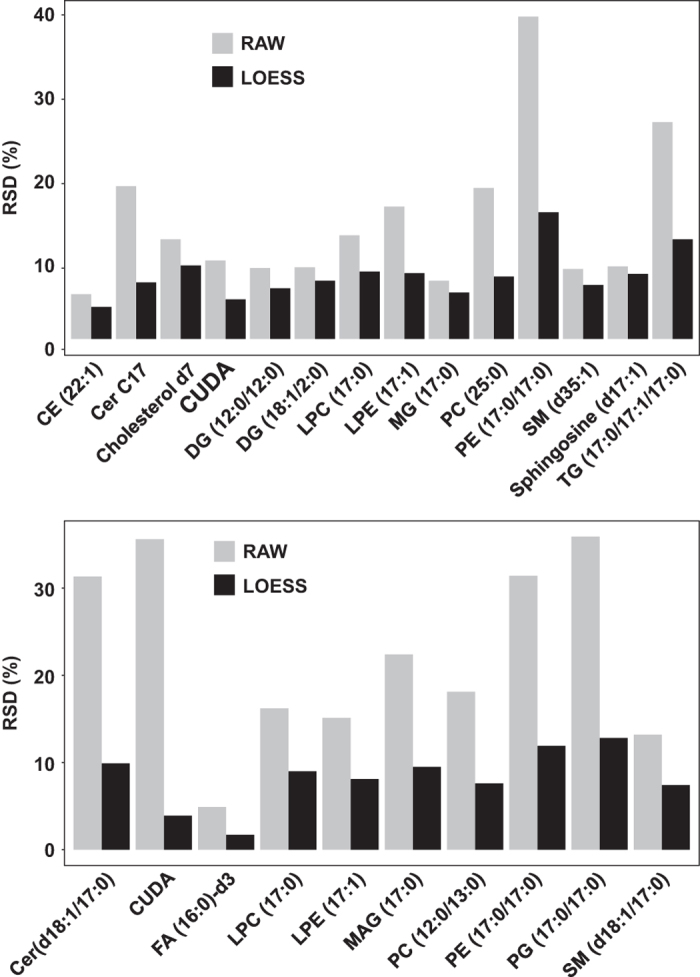
Batch effect removal using LOESS lowered the analytical variability. Relative standard deviation (RSD %) for internal standards across all samples. Upper panel: ESI positive mode; lower panel: ESI negative mode.

To calculate the quality of the dataset generated by the lipidomics workflow, we computed the relative standard deviations of the intensity levels of the internal standards, as these were not used in the normalization scheme. Fig. 3 shows these values for positive and negative electrospray ionization modes before and after LOESS normalization. All internal standards showed reproducibility better than 20% RSD, proving that the data acquisition and data processing method has produced a high-quality lipidomics dataset.

**Figure 4:**
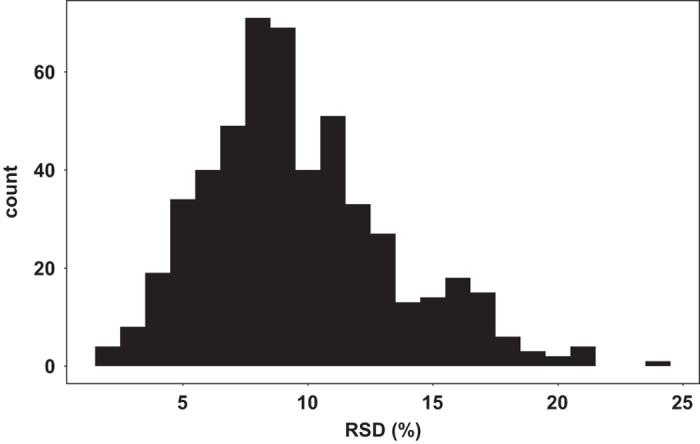
Reproducibility of peak heights for the detected compounds in QC samples. Histogram shows the distribution of RSD (%) for compounds in the QC samples.

To show the technical reproducibility of the lipidomics assay, we have accessed the RSDs of measured lipids in the QC samples. Levels of high quality lipids should not change much in quality control samples, providing evidence our data processing methods are robust and reproducible. Fig. 4 shows the histogram of these RSDs. In the same manner as the internal standards (Fig. 3), we see that more than 95% of all lipid species were detected at a reproducibility of better than 20% in the QC samples, highlighting the excellent quality of the data. Consequently, we removed lipids from the data set that showed technical errors of more than 25% RSD. Finally, a paired *t*-test analysis of the replicated samples (*n* = 20) shows that up to 80% of the lipid species were not significantly different (*p* > 0.05), showing the removal of the batch effect by the data processing pipeline and the low analytical variability in this dataset. Results of RSD in QC samples and *p*-values of paired t-test in duplicate samples are provided in the data record (adni1_qcrsd_ttest.csv).

**Figure 5:**
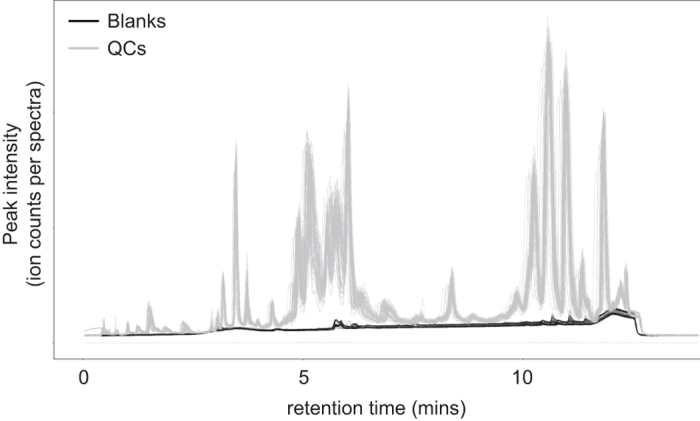
Quality of the LC-MS analyses for ADNI1 specimens. Minimum carryover was observed in the negative control samples analyzed across the study. Overlaying total ion chromatograms (TIC) plot of QCs (grey lines) and all blanks (black lines) for all the batches

As demonstrated for Fig. 5, we detected no discernible carry-over effect when overlaying blank and sample total ion chromatograms. The same quality was found when investigating the data for extracted ion chromatograms, including triacylglycerides or cholesteryl esters.

## Usage Notes

Investigators using the ADNI metabolomics data are subject to the general ADNI data usage access policies and user agreements. Details on how to apply for access, and usage rules can be found at the ADNI website: http://adni.loni.usc.edu/data-samples/access-data/. ADNI is one the largest cohort studies on Alzheimer’s disease. It is funded by National Institute Aging as one of their flagship studies with a mandate that all the acquired datasets will be made publicly available without a wait for a formal publication. It is the only human cohort study that has provided genetics, imaging, clinical, medical, pathological and biomarkers datasets in a single repository and have made it publicly available, enabling combined analysis of these heterogenous datasets. It has ensured the use of single subject identifier across all datasets, enabling a seamless data merging. All the data have been submitted to ADNI repository with a quality control report and a data dictionary so new users can understand the measured variables without contacting the original data submitters. ADNI study sets an unprecedented example for how large human cohorts studies with heterogenous datasets can be catalogued and publicly shared to enable new analyses that goes beyond one research group. Because ADNI is a human cohort study with available genetics and other medical datasets, a new user need to first register and agreed to the term of fair use of data and the code of conduct. There are no repository including metabolomics workbench or metabolights that can accept these other datasets such as whole genome sequencing data, MRI-scans, blood proteomics and medication data for a human cohort study. Therefore, we have decided to submit the raw LCMS data files and all the processed results into ADNI and Sage Bionetwork repositories. Several additional files were generated for the lipidomics assay which could not be submitted to the ADNI repository, we have provided them at the Sage Bionetwork repository for new users. To confirm with the ADNI’s data usage terms, a new user also need to register at the Synapse website to access these files. First, a new user need to register at the http://adni.loni.usc.edu/data-samples/access-data/ to get access to the ADNI data. Then a user need to register at the Synpase.org to get access to the additional files at the project page at https://www.synapse.org/#!Synapse:syn9993525. To access the ADNI data user need to go to the https://ida.loni.usc.edu/pages/access/studyData.jsp. The page lists genetics, imaging, clinical and biomarkers datasets for the ADNI study. Because both databases are managed by different entities, users must register for separate user accounts for ADNI and Synapse respectively.

## Consortia

*Alzheimer’s Disease Neuroimaging Initiative*

A complete listing of ADNI investigators can be found at: http://adni.loni.usc.edu/wp-content/uploads/how_to_apply/ADNI_Acknowledgement_List.pdf

*Alzheimer’s Disease Metabolomics Consortium*

A list of members is available at: https://sites.duke.edu/adnimetab/files/2017/06/ADMCTeamMembersJun14.pdf

## Additional Information

**How to cite this article**: Barupal, D. K. *et al*. Generation and quality control of lipidomics data for the alzheimer’s disease neuroimaging initiative cohort. *Sci. Data*. 5:180263 doi: 10.1038/sdata.2018.263 (2018).

**Publisher’s note**: Springer Nature remains neutral with regard to jurisdictional claims in published maps and institutional affiliations.

## Supplementary Material



## Figures and Tables

**Table 1:** Baseline demographics and clinical data of studied ADNI subjects as determined at baseline.

	CN (*n* = 226)	MCI (*n* = 392)	AD (*n* = 188)	*p*-value
Age (years)	75.5 (72.2-78.4)	75.1 (70.1-80.4)	75.8 (70.8-80.5)	0.29
Gender (% Male)	47.8%	35.4%	47.6%	0.0019
APOE ε4 (%)	26.5%	53.3%	66.1%	<0.0001
MMSE	29.0 (29.0-30.0)	27.0 (26.0-28.0)	23.0 (22.0-25.0)	<0.0001
ADAS-Cog13	9.33 (6.0-12.3)	18.3 (14.7-23.0)	28.5 (23.7-34.0)	<0.0001
APOE- Apolipoprotein E; MMSE- Mini–Mental State Examination; ADAS-Cog 13 Alzheimer’s Disease Assessment Scale cognitive scale, 13-item version. P-values are based on Chi-square test for APOE status and Kruskal-Wallace for all other variables. P-values were not corrected for multiple testing				

**Table 2:** Lipid ion database used the West Coast Metabolomics Center for generation of lipidomics data matrices.

	ESI( + )		ESI(–)	
	Known	Unknown	Known	Unknown
Target ions	423	92	252	205
Unique compounds	286	92	196	205
Internal standards	14	0	11	0

**Table 3:** Description of data records.

File Name	Description	Type	Location	URL
**Primary Files**				
ADMC_ADNI1_LIPIDOMICS.csv	Normalized & filtered lipidomics dataset. First column in this file is the RID and rest of the columns are lipid ids.	Data	LONI	http://dx.doi.org/10.7303/syn10208594.1
ADNI_ADMCLIPIDOMICS_DICT.csv	Data dictionary for lipid species. It has the ionization mode, adduct information, precursor ion value, retention time and InChiKey values.	Data Dict	LONI	http://dx.doi.org/10.7303/syn10208614.1
**ADMC Supplemental Materials**				
ADNI1_LIPIDOMICS_RAW_DATA_ESI_POS.zip	Raw LC-MS data files (ESI + mode) in the Agilent (.d) format.	Data	LONI	http://dx.doi.org/10.7303/syn10495782.1
ADNI1_LIPIDOMICS_RAW_DATA_ESI_NEG.zip	Raw LC-MS data files (ESI -mode) in the Agilent (.d) format.	Data	LONI	http://dx.doi.org/10.7303/syn10495771.1
wcmc-lipidomics-database.zip	Curated database of plasma lipid ions for ESI ( + /-) modes. It has m/z values, adduct information and retention time for the targets.	Metadata	Synapse	http://dx.doi.org/10.7303/syn10208660.1
adni1-lipidomics-acq.zip	Agilent Qual 7.0 method that used for the data acquisition on the LCMS instrument.	Method	Synapse	http://dx.doi.org/10.7303/syn10208671.1
adni1-lipidomics-eics.zip	Extracted Ion Chromatograms in .csv format for ESI ( + /-) modes. These files were generated by the Agilent Qual software.	Data	LONI	http://dx.doi.org/10.7303/syn10495758.2
adni1-lipidomics-script.r	R-script for data generation and quality control	Script	Synapse	http://dx.doi.org/10.7303/syn10208679.1
adni1_acqtime.zip	Details about sample acquisition time and labels. It is required by the R-script	Method	Synapse	http://dx.doi.org/10.7303/syn10208662.1
adni1_qcrsd_ttest.csv	RSDs in QC samples and the *p*-values (student *t*-test paired) for duplicates samples	Data	LONI	http://dx.doi.org/10.7303/syn10208665.3
adni1_lipidomics_data_processing.zip	Input, intermediate and output data matrices used by the R-script.	Data	Synapse	https://www.synapse.org/#!Synapse:syn12973205
adni1_lipidomics_isa_tab.zip	ISA-tab file for the RID to raw LCMC data files mapping	Data	Synapse	https://www.synapse.org/#!Synapse:syn12972536
adni1_loess_results.zip	PCA plots for before and after the LOESS normalization	Data	Synapse	https://www.synapse.org/#!Synapse:syn12973204
**Clinical Data**				
ADNI_All_Clinical_Data_16May2016.csv	Clinical variables (a subset of ADNI’s complete list) snapshot from May, 2016	Data	LONI	http://dx.doi.org/10.7303/syn7477271.1
Fasting Status.txt	Fasting status of participants at time of blood draw	Data	Synapse	http://dx.doi.org/10.7303/syn9774830.1
ADNI Key Clinical Variables Subset Data Dictionary.xlsx	Data dictionary for a key subset of variables in ADNI_All_Clinical_Data_16May2016.csv (for full version see “Data Dictionary [ADNI1,GO,2] (DATADIC.csv)” on LONI)	Data dict	Synapse	http://dx.doi.org/10.7303/syn9758900.1
